# Poisonous Plants of the Indian Himalaya: An Overview

**DOI:** 10.3390/metabo12060540

**Published:** 2022-06-13

**Authors:** Abhishek Jamloki, Vijay Laxmi Trivedi, M. C. Nautiyal, Prabhakar Semwal, Natália Cruz-Martins

**Affiliations:** 1 High Altitude Plant Physiology Research Centre (HAPPRC), H.N.B. Garhwal University, Srinagar Garhwal 246174, India; abhijamloki@gmail.com (A.J.); vijaylaxmitrivedi@gmail.com (V.L.T.); mcnautiyal@rediffmail.com (M.C.N.); 2 Department of Life Sciences, Graphic Era (Deemed to be University), Dehradun 248002, India; 3 Faculty of Medicine, University of Porto (FMUP), 4200-319 Porto, Portugal; 4 Institute for Research and Innovation in Health (i3S), University of Porto, 4200-135 Porto, Portugal; 5 Institute of Research and Advanced Training in Health Sciences and Technologies (CESPU), Rua Central de Gandra, 1317, 4585-116 Gandra, Portugal; 6 TOXRUN—Toxicology Research Unit, University Institute of Health Sciences, CESPU, CRL, 4585-116 Gandra, Portugal

**Keywords:** Indian Himalaya, pharmaceuticals, poisonous plants, bioactive compounds, toxicity

## Abstract

Indian Himalayan region (IHR) supports a wide diversity of plants and most of them are known for their medicinal value. Humankind has been using medicinal plants since the inception of civilization. Various types of bioactive compounds are found in plants, which are directly and indirectly beneficial for plants as well as humans. These bioactive compounds are highly useful and being used as a strong source of medicines, pharmaceuticals, agrochemicals, food additives, fragrances, and flavoring agents. Apart from this, several plant species contain some toxic compounds that affect the health of many forms of life as well as cause their death. These plants are known as poisonous plants, because of their toxicity to both humans and animals. Therefore, it is necessary to know in what quantity they should be taken so that it does not have a negative impact on health. Recent studies on poisonous plants have raised awareness among people who are at risk of plant toxicity in different parts of the world. The main aim of this review article is to explore the current knowledge about the poisonous plants of the Indian Himalayas along with the importance of these poisonous plants to treat different ailments. The findings of the present review will be helpful to different pharmaceutical industries, the scientific community and researchers around the world.

## 1. Introduction

The documented use of plants is as old as the existence of human beings on earth [1], for both feeding and healing purposes [2,3]. As per the survey report published by the World Health Organization (WHO), it is estimated that about 80% of the population from developing countries depend on traditional medicine for primary health care [4,5,6,7].

Medicinal plants are composed of a plethora of secondary metabolites, such as alkaloids, phenolics, flavonoids, terpenoids and glycosides, which act to protect them from adverse situations [8,9,10,11,12]. Most plant products are biologically and pharmacologically useful because of their therapeutic properties, while others are toxic to both humans and animals due to the presence of harmful by-products [1]. These plants are known as poisonous plants, because of their toxic nature, and are widely distributed around the world, being used by indigenous people for hunting, fishing and the treatment of different diseases [13,14].

**Figure 1:**
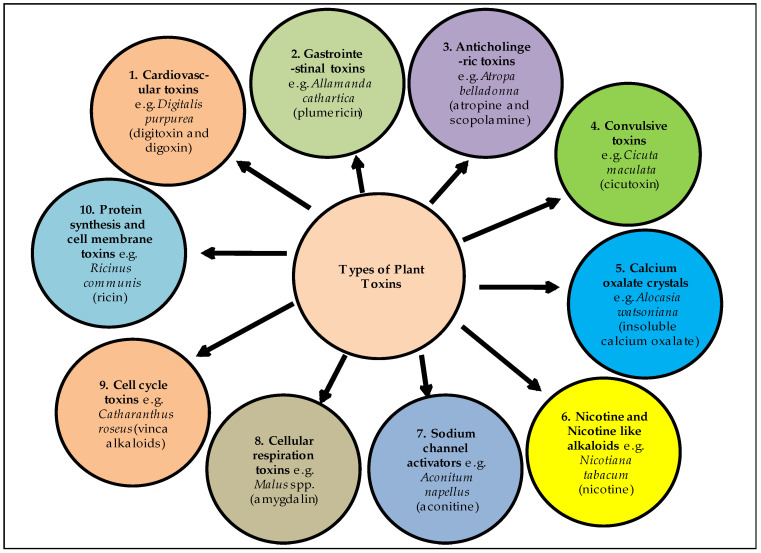
Types of plants toxins and their mode of actions.

The toxic nature of a plant species vary from species to species, and depends on several factors, including chemical, physical, biological and environmental (presence of chemical substances, its concentration, age of plant, used part, ripening state of its fruits, soil type, temperature, humidity, etc.) [15]. The poisoning may result either from contact, which may cause skin irritation; ingestion, which may result in internal toxicity; absorption; or inhalation through the respiratory tract [16]. Plant toxins can be divided into several groups, such as gastrointestinal toxins, cardiovascular toxins, convulsive toxins, anti-cholinergic toxins, nicotine and nicotine-like alkaloids, calcium oxalate crystals and cellular respiration toxins [17] (Figure 1).

**Figure 2:**
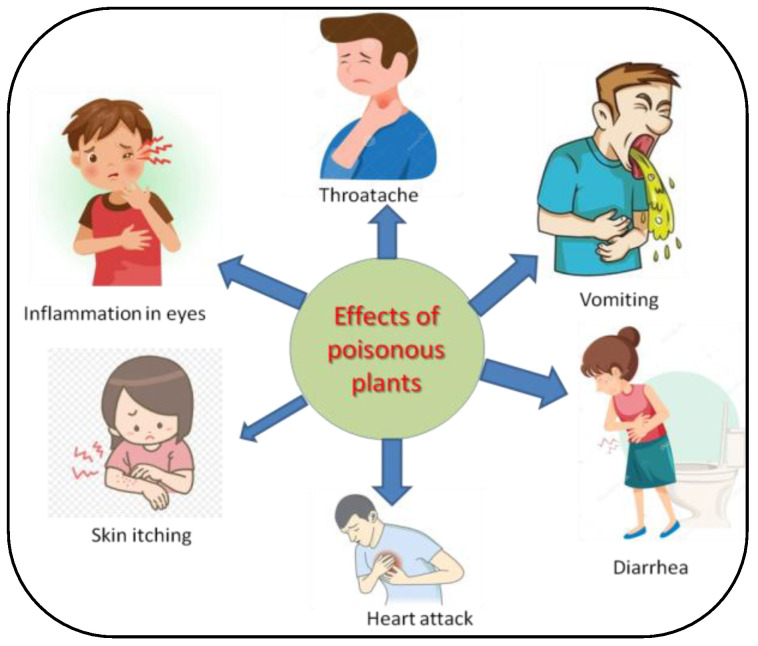
General symptoms of the poisonous plants in humans after touching and ingestion.

Most poisonings cases are characterized by irritations of the gastrointestinal tract, such as vomiting, nausea or severe diarrhea, and others by dermatological discomfort, such as dermatitis (Figure 2). However, there are more severe cases of poisonings, in which the central nervous system or cardiorespiratory function can be affected, and death can even occur [18]. Plant toxins are closely related to human and animal health aspects [19], and some toxic compounds might even be applied as effective treatments for human diseases [20].

The toxicity of poisonous plants in some cases resides in whole plant, while in other cases in some parts of the plant, such as the shoot, leaves, flower, seeds, bark or even latex [21]. The continuous research and development in plant knowledge has promoted a marked increase in the awareness and usefulness of plants for medicinal purposes [22], as well as on their toxicological profiles [23,24].

Most individuals are not familiar with the toxicity of most plants found around them, an aspect that is potentially harmful if they establish a direct contact or even ingest them. In animals, most poisonous plants cause poisoning when they are accidently grazed by them [25]. Despite local elderly people passing the knowledge of poisonous plants from one generation to the next one, it is very important to provide general awareness regarding their toxicological profiles [26]. Thus, the best approach for minimizing accidental intoxication with poisonous plants is to make people aware of plants’ toxicity and their harmful effects on them and other animals. This information should be dispersed in general by the population, childhood educators and official entities that together can play a special role in this sense.

The purpose of this review is to explore the current knowledge about the poisonous plants of the Indian Himalayas, as well as the compounds found in these plants that are responsible for their toxicity to humans and other animals and to provide an overview on the medicinal properties of these plants for healing human diseases.

## 2. Methodology

**Figure 3:**
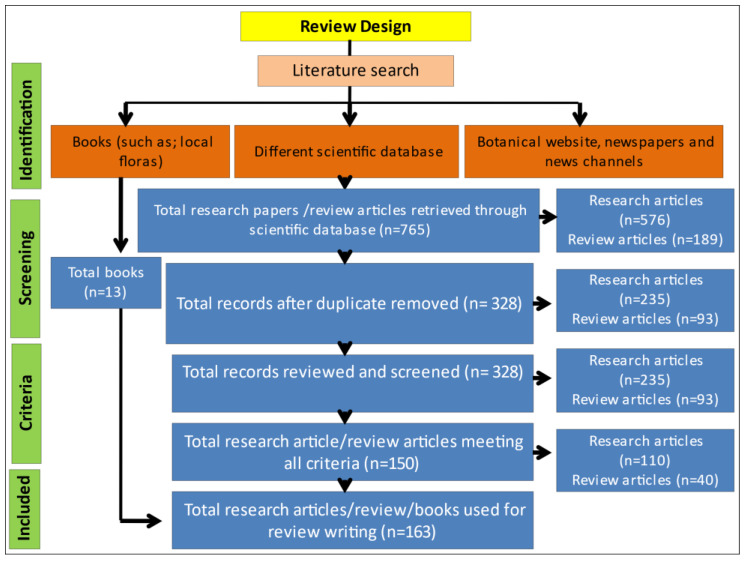
Flowchart for study design.

A comprehensive and effective review was systematically prepared on the basis of information available in research papers of various electronic-based journals and books, which enhances its novelty. To design the comprehensive review, we first collected information on the following study questions: (1) How many poisonous plants are reported in the Indian Himalayas and what causes their toxicity? (2) What are the toxicity symptoms after ingestion? (3) How these plants are useful for human welfare. In order to search the literature related to this study, various search engines were used, including Web of Science, Google Scholar, EBSCO Green FILE, Research gate and PubMed. To find research papers related to the present study the following keywords were used: “*poisonous plants of Himalaya”* or “*toxicity of poisonous plants”* or “*toxic compounds*”. Certain criteria were set for the screening and review process, such as: (1) the article presents original primary research and not a review or analysis of secondary data; (2) it is peer-reviewed; (3) it is published in the English language; (4) the full text of the article should be available online in various scientific search engines. A total of 163 manuscripts/books were finalized from 1981 to March 2022 to write this review, which includes original research papers, review papers, book chapters and case studies. For the initial design of this review, several books of local flora, news channels and newspapers were searched. For information on plants synonyms and their distribution range, some botanical websites such as; www.theplantlist.org, www.plantoftheworldonline.org, www.gbif.org, www.jstor.org and www.tropics.org (all accessed on 22 May 2022) were also used. The complete methodology of this study has been presented in Figure 3.

## 3. Poisonous Plants of Indian Himalaya

The Indian Himalayan region (IHR) homes a huge variety of plant species that have been used in several ways, i.e., emergency food/nutraceutical, medicinal, pharmaceutical purposes, etc. [27]. The IHR has a large diversity of plants and most of them are known for their medicinal properties, with some of them being known for their toxicity. Poisonous plants of the IHR produce a variety of toxins that have negative effects on human and animal health, ranging from mild allergies to serious medical complication and even death. In India, it is estimated that more than 50,000 people die from toxic exposure every year, which is the highest number in the world, and plants account for 1.7% of all toxic exposures [28], and are mostly used for robbery and suicidal purposes. Poisoning is the fourth most common cause of mortality in India [29]. Poisonous plants most often affect grazing livestock, which is a major concern for both farmers and veterinarians. Grazing is considered a common routine in livestock management, but it exposes animals to a variety of poisonous plants, especially when there is a lack of fodder availability [30]. Animals that are already experiencing nutritional stress are more vulnerable to plant toxicity. The Indian subcontinent has the biggest population of the livestock in the world, accounting for about 7% of its income [31]. Sometimes, the lack of knowledge and unawareness from inhabitants means that they use these plants for food, fodder and medicinal purposes, or are even subjected to accidental exposure, which is sometimes life-threating to both humans and animals. Poisonous plants are harmful for livestock and causes of economic loss to the livestock sector. Some of the major poisonous plants of the IHR are further described below (Table 1).

### 3.1. Abrus precatorius *Linn.*

The seeds of *Abrus precatorius* (Indian licorice, Fabaceae) are highly toxic and contain some active compounds, such as abrine, abrasine, abraline, abrin, abricin, abrusgenicacid, etc. [32]. Abrin is more toxic than the other active compounds, which is a toxalbumin that inhibits protein synthesis and causes cell death [33]. Even consuming one of its seeds can be fatal for both children and adults. The lethal dose or (LD_50_) of abrin toxin for human is 0.1–1 µg/kg body weight [34].

### 3.2. Aconitum ferox *Wall. Ex Ser.* (Syn Aconitum Atrox *Walp*)

*Aconitum ferox* (Indian aconite, Ranunculaceae), is an erect and perennial herb whose distribution ranges from temperate to alpine regions of India, Nepal, Bhutan and China [35,36,37]. It is a rhizomatous poisonous herb, but with great pseudaconitine, veratroylbikhaconine, medicinal properties when used after vigorous purification and in the right amounts. Bikhaconitine, veratroylpseudaconine, norditerpenoid alkaloids and quinolinones are some active compounds present in the whole plant [38,39]. The plant is used as poison for arrow heads [40]. The lethal dose of aconitine in human is 2 to 6 mg/kg body weight [41].

### 3.3. Aconitum hookeri *Stapf.*

*Aconitum hookeri* (Hooker’s Monkshood, Ranunculaceae) extends its distribution from India, Nepal, Bhutan and China [37]. The whole plant is reported as poisonous, but its rhizomes are used as a medicine [42].

### 3.4. Aconitum lethale *Griff.* (Syn Aconitum Balfourii *Stapf.*)

*Aconitum lethale* (Balfour’s Monkshood, Ranunculaceae) is an erect glabrous herb, whose main distribution is in Himalayan region. Aconitine, pseudoaconitine [43], balfourine [44,45], norditerpenoid alkaloids [46] are some active compounds found in this species. The whole plant is reported as toxic for humans and other animals.

### 3.5. Aconitum napellus *Linn.*

*Aconitum napellus* (Violet Monkshood, Ranunculaceae) is a biennial plant, with geminate tubers. Stems are usually simple, erect in the lower part, glabrous or hairy [47]. It is generally distributed in the Himalayan region of Nepal, India, China, and Pakistan [48]. The primary toxin of *A. napellus* is aconitine, which is distributed throughout the plant, but its concentration is highest in its roots and leaves [17].

### 3.6. Aconitum spicatum (Brühl) *Stapf.*

*Aconitum spicatum* (Nepal Aconite, Ranunculaceae) is a shrub species, usually sparsely pubescent and simple [36]. The root tubers of this species contain some active compounds such as aconitine, mesaconitine, bikhaconitie, deoxyaconitine, hypaconitine, spicatine A and B [49]. *A. spicatum* is highly toxic and used as arrow poison in Nepal Himalaya [50].

### 3.7. Aquilegia pubiflora *Wall. Ex Royle*

*Aquilegia pubiflora* (Himalayan Columbine, Ranunculaceae) is another important herb widespread in the Himalayan region of India, Pakistan and Afghanistan [51]. Isovitexin, isoorientin, vitexin, chlorogenic acid, orientin, cumeric acid, sinapic acid, ferulic acid are some active compounds found in this species [52]. 

### 3.8. Aesculus indica (*Wall. Ex Camb.*) *Hoof. f.*

*Aesculus indica* (Indian horse-chestnut or Himalayan horse chestnut, Hippocastanaceae) is widely distributed in low-temperature regions of the world, and is commonly found in North Western Himalaya in the Indian context [53]. *A. indica* is large sized deciduous and perennial tree species that attains a height of up to 20 m. It is widely used in traditional medicine systems to treat many diseases. *A. indica* is poisonous to humans and other animals due to the presence of a saponin-class toxin called escin or aesculin [54,55]. After ingestion, aesculin enters the blood and destroys red blood cells. The young leaves and flowers of this plant species are more toxic than mature leaves. The bark and seeds also contain small amounts of aescin [55]. *A. indica* poisoning can cause fatigue, paralysis, coma, and even death. The lethal dose or LD_50_ was observed to be 10.6 mg/g body weight for chicks with a single dose of the seed extract (*A. indica*) and 10.7 mg/g body weight with hamster. Administration of *A. indica* for 2 consecutive days showed 6.5 mg/g LD_50_ [54].

### 3.9. Cannabis sativa *Linn.*

*Cannabis sativa* (Hemp or bhang, Cannabaceae) is one of the most important industrial crops distributed at global level [56] for its psychoactive resins. The native distribution of the species is in Central Asia, Siberia, China and the Himalayas [56]. *C. sativa* contains more than 400 active compounds, but the major psychoactive toxic constituents are 9-tetrahydrocannabinol (THC) and cannabidiol (CBD) [27]. The lethal dose or LD_50_ of THC is not determined in humans, but in cattle, it was observed to be 40 to 130 mg/kg body weight [57].

### 3.10. Convallaria majalis *Linn.*

*Convallaria majalis* (Lily of the valley, Asparagaceae) is an herbaceous plant native from Europe that forms extensive colonies by spreading underground rhizomes. It is considered to be the most potent cardiotoxic plant due to the presence of approximately 38 cardiac glycosides (cardiotoxins), such as convallatoxin, convallarin and convallamarin, etc. [58,59]. This plant is highly poisonous for humans and other animals, causing severe cardiac disturbances [60]. The lethal dose or LD_50_ of convallatoxin was observed to be 0.08 mg/kg body weight [61].

### 3.11. Delphinium brunonianum Royle

*Delphinium brunonianum* (Musk larkspur, Ranunculaceae) is a high-altitude plant, native from China and distributed in the western Himalayas [62]. The species contains diterpenoid alkaloids and methyllycaconinite, which have been used for poisonous and medicinal purposes [27]. The lethal dose or LD_50_ of *Delphinium* spp. for cattle was observed to be 25–40 mg/kg body weight and it may depend on the presence of alkaloid compounds [63]. 

### 3.12. Digitalis purpurea *Linn.*

*Digitalis purpurea* (Lady’s glove or common foxglove, Plantaginaceae) is a biennial or perennial herb (1–2 m tall), distributed in Western Europe, northwestern Africa and Asia. It contains several active compounds i.e., glycosides, digitoxin, aglycone gitoxigenin, gitoxin, digitonine and anthraquinones. The primary toxins are digitoxin and digoxin, present throughout the plant [17]. The LD_50_ of digitoxin was 0.18 mg/kg for cats and 60 mg/kg body weight for guinea pigs [64].

### 3.13. Eupatorium adenophorum *Spreng.*

*Eupatorium adenophorum* (Sticky snakeroot or Crofton weed, Asteraceae) is a perennial herbaceous invasive plant species native to Mexico and Central America. *E. adenophorum* is used in Ayurveda and Chinese medicine for the treatment of wounds, fever, diabetes, dysentery and jaundice [65]. *E. adenophorum* has pneumotoxic and hepatotoxic effects on animals, especially horses being more susceptible to its toxicity. Consumption of leaves of this plant by horses causes a chronic pulmonary disease known as Numinbah Horse Sickness, whereas in goats no effect has been observed after ingestion in the Nepal Himalayas [66,67]. It causes anorexia and photosensitization in cattle. 2-deoxo-2-(acetyloxy)-9-oxoageraphorone, 9-oxo-10, 11-dehydroageraphorone 10Hβ-9-oxoageraphorone, and 10Hα-9-oxo-ageraphorone are some hepatotoxic compound present in Crofton weed [68] and cause hepatotoxicity in mice and rats [69,70]. 2-deoxo-2-(acetyloxy)-9-oxoageraphorone (215–4640 mg/kg body weight, orally) showed the lowest LD_50_ at 926 mg/kg body weight in male mice in contrast with 9-oxo-agerophorone (1470 mg/kg body weight) and 9-oxo-10,11-dehydro-agerophorone (1470 mg/kg body weight) [71].

### 3.14. Heracleum canescens *Lindl.*

*Heracleum canescens* (Grey-Hairy Hogweed, Apiaceae) is an herbaceous plant species that is found from the Kashmir to Nepal Himalayas. The height of the plant is 30–70 cm and its leaves are compound, ovate and covered with white hairs. *Heracleum* spp. contains a phytotoxic compound furanocoumarin in their roots, leaves, stem, flower and fruits. Skin contacts with furanocoumarins caused sensitization when exposed to sunlight or UV light. Furanocoumarins enters the nucleus and binds with DNA, and causes cell death and inflammation [72].

### 3.15. Hyoscyamus niger *Linn.*

*Hyoscyamus niger* (Henbane or Khurasaniajwain, Solanaceae) is an annual, biennial or perennial herb distributed in Asia, Africa and Europe [73]. All plant parts comprise alkaloids which are toxic, viz. atropine, hyoscyamine, scopolamine and tropane, that act as broncho-dilators, urinary bladder relaxants, having antisecretory, spasmolytic, hypnotic, pupil dilating, hallucinogenic and sedative effects, etc. Henbane is toxic to cattle, wild animals, fish, and birds [27]. A fatal dose of atropine to humans is greater than 10 mg, whereas scopolamine is toxic at 2–4 mg [74]. 

### 3.16. Lantana camara *Linn.*

*Lantana camara* (wild or red sage, Verbenaceae) is invasive plant species and native to the tropical region of Africa and America [75]. It is erect or sub-scandent woody perennial shrub with 0.3–1.8 m or more heigh. *Lantana* was introduced as an ornamental shrub at Calcutta Botanical Garden, India in the year 1809 [76]. The leaves of *L. camara* are poisonous to animals and cause the poisoning of cows, buffalo, goats and sheep. Lantadenes is a pentacyclic triterpenoid present in its leaves and cause photosensitivity and hepatotoxicity in grazing animals [77]. The sub-acute toxicity dose of lantadenes in the guinea pig is 25 mg/kg body weight [78].

### 3.17. Melia azedarach *Linn.*

*Melia azedarach* (Chinaberry tree or pride of India or bead-tree, Meliaceae) is a woody plant species that is highly toxic to humans and other animals. The whole plant is toxic and eating even several berries can result in death. Meliatoxins are an active compound found in *M. azedarach*, and are responsible for its toxicity [27,79]. However, mature berries are more toxic than young ones. A single dose of green leaves around 30 g/kg body weight and fruit 5 g/kg body weight is lethal for cattle and death within 17–48 h after ingestion [80,81], while the oral lethal dose (LD_50_) of meliatoxin in pigs is 6.4 mg/kg body weight [82].

### 3.18. Rhododendron campanulatum *D. Don*

*Rhododendron campanulatum* (Bell Rhododendron, Ericaceae) is a shrub distributed from the tree-line ecotone, timberline and subalpine forests of India, Nepal, Bhutan and China [83]. The flowering buds of this species are toxic for cattle [84]. Andromedotoxin is a toxic compound present in this species, which causes salivation, diarrhea, loss of energy and finally death of livestock [85]. Grayanotoxin analogues are detected (30 ppm) in local honey partly derived from two species of rhododendron (*R. arboreum* and *R. campanulatum*), being able to trigger poisoning symptoms in humans, as the lethal dose (LD_50_) in mice is 0.87 to 1.3 mg/kg body weight [86,87]. Fresh leaves can be toxic in the amount of 0.1% of the goat’s body weight [88]. On the other hand, grayanotoxin toxicity in humans is caused by the ingestion of a minimum of 10 g of contaminated honey [89].

### 3.19. Ricinus communis *Linn.*

*Ricinus communis* (Castor bean or castor oil plant, Euphorbiaceae) is a soft wooden small tree developed throughout tropics and warm temperature regions [90]. This plant is indigenous to the southeastern Mediterranean Basin, Eastern Africa and India but is widespread throughout tropical regions and is widely used as an ornamental plant [91]. It is classified as the most poisonous plant on Earth for humans [92] and its toxic nature is due to the presence of ricin [92] in the seeds of this species [93], which is a type 2 ribosome-inactivating protein. In medical reports, depending on the number of seeds ingested, this plant can cause mild to severe symptoms, including a fatal outcome, ranginf from an uptake of only single seeds to up to 30 seeds [94]. The estimated lethal dose of ricin toxin to humans is 1–10 µg/kg body weight following inhalation or injection [95].

### 3.20. Solanum xanthocarpum *Schrad. & H. Wendl.*

*Solanum xanthocarpum* (Yellow Berried Night Shade, Solanaceae) is distributed throughout India, and known as its high medicinal value. *S. xanthocarpum* is a herbaceous plant species and attains a height of up to 50–70 cm. Fruits of this plant species contain steroidal glycoalkaloids such as solasonine and solamargine [96], which cause toxicity in animals and human [97]. The lethal dose (LD_50_) of solamargine in rats was observed to be 42 mg/kg body weight, while no toxicity was recorded at doses below 35 mg/kg body weight (LD_50_) [98].

### 3.21. Taxus baccata *Linn.*

*Taxus baccata* (English yew or European yew, Taxaceae) is an evergreen, dioecious tree and grows up to 25 m tall. It is distributed from Europe to the eastern Himalayas of Asia. This plant species is known for its anti-cancer activity. Taxines are the active, poisonous constituents in this species and cause toxicity in animals and humans [99]. After ingestion, it increased Ca^2+^ in cytoplasm but inhibited Na^+^ and Ca^2+^ channels and caused cardiac failure [100]. The lethal doses (LD_50_) of taxine in different animals were recorded as 19.72–21.88 mg/kg body weight for mice, 20.18 mg/kg body weight for rats [101], 3.5 mg/kg body weight for rabbits, 0.2 to 0.5% for ruminant and 0.05% body weight for horses, respectively [99,102].

### 3.22. Silybum marianum *Gaertn.*

*Silybum marianum* (Milk thistle, Asteraceae) is an annual or biennial herb whose therapeutic history dates back to 2000 years ago and was used to treat different ailments [103]. This plant species is native from Asia and Southern Europe, but now it is found throughout the world [104]. This species is highly toxic for cattle due to the presence of potassium nitrate (KNO_3_) in plant materials: after the ingestion of plant material by cattle, potassium nitrate is broken down into nitrite ions by bacteria present in cattle stomachs. Nitrite ions then combines with hemoglobin and produce methemoglobin, which blocks the transport of oxygen and may cause respiratory distress [105].

**Table 1:** Poisonous plants of Indian Himalayas.

S. No.	Plant Species	Family	Toxic Compound	Symptoms	Reference
1	*Abrus precatorius* Linn.	Fabaceae	Abrin	In humans, it causes vomiting, nausea, difficulty in swallowing, throat pain, high fever, weakness irritation in eyes, severe diarrhoea and even death. After ingestion by livestock it causes nasal discharge, salivation, severe diarrhoea, abortion and eventual death in pregnant animals.	[14]
2	*Aconitum chasmanthum* Stapf ex Holmes	Ranunculaceae	Aconitine, diterpenoid alkaloid	Cardiotoxins and neurotoxins, skin contact cause numbness.	[27,106]
3	*Aconitum ferox* Wall. ex Ser.	Ranunculaceae	Pseudoaconitine and bikhaconitine	Cardio and neurotoxicity.	[38,39]
4	*Aconitum lethale* Griff.	Ranunculaceae	Pseudoaconitine and aconitine, balfourine	Cardio and neurotoxicity.	[43,44]
5	*Aconitum laeve* Royle	Ranunculaceae	8-methyllycaconitine, 14-demethyllycaconitine, and N-deethyllycaconitine-N-aldehyde	Cardio and neurotoxicity.	[107,108]
6	*Aesculus indica* (Wall. Ex Camb.) Hoof. f.	Hippocastanaceae	Escin or aesculin	After consumption it causes gastro-intestinal problems, dizziness, nausea, vomiting, headache, fatigue and pruritus, while excessive consumption may cause paralysis and death.	[54]
8	*Ageratum conyzoides* Linn.	Asteraceae	Pyrrolizidine alkaloids	Due to contact with the plant, it causes skin problems such as as itching and rashes in susceptible individuals. Animals usually avoid browsing it, but accidental consumption causes very high fever, diarrhoea, anorexia and finally death within few hours.	[109]
9	*Aloe vera* (L.) Burm.f.	Xanthorrhoeaceae	Aloin or barbaloin an anthraquinone glycoside	Excessive consumption may cause nausea, abdominal pain, vomiting, hyperkalemia and cardiac dysrhythmias.	[17]
10	*Anagallis arvensis* Linn.	Primulaceae	Primin	Consumption of the plant causes an acute headache, nausea, unconsciousness, anorexia, body pains, general weakness, bloody diarrhoea, sudden drop in body temperature and eventually death.	[14,110]
11	*Aquilegia pubiflora* Wall. ex Royle	Ranunculaceae	Isovitexin, isoorientin, vitexin, chlorogenic acid, orientin, cumeric acid, sinapic acid, ferulic acid	Cardiogenic toxins cause gastroenteritis and heart palpitations.	[111]
12	*Argemone Mexicana* Linn.	Papaveraceae	Sanguinarine and dihydrosanguinarine alkaloids present in Argemone oil.	Seeds are toxic and cause nausea, intense headaches, vomiting, severe diarrhoea, oedema of legs and feet.	[14,112]
13	*Arisaema tortuosum* (Wall.) Schott	Juncaceae	Raphide (Calcium oxalate)	Intake of tubers causes irritation of the skin and mucous membrane, mouth and body pain, slow breathing and suffocation.	[113]
14	*Arisaema triphyllum* (L.) Schott	Araceae	Raphide (Calcium oxalate)	Irritation of the skin and the mucous membrane and body pain.	[113]
15	*Artemisia nilagirica* (C.B. Clarke) Pamp.	Asteraceae	Lactones	Ingestion of large doses by animals causes headaches, nausea, vomiting and abortion of pregnant animals as a result of contraction of the uterus.	[14,114]
16	*Atropa belladonna* Linn.	Solanaceae	Atropine and Scopolamine	Plant ingestion may cause vomiting, nausea, diarrhea and abdominal cramps.	[17]
17	*Calotropis procera* (Aiton) W.T.Aiton	Asclepiadaceae	Uscharin, Calotoxin, Calotropin, Calactin, and Calotropage	The milky latex of this plant act as the skin and mucous membranes irritant, that causes blisters in both humans and animals. Accidental exposure to latex can cause eye swelling and redness. Both the leaves and the latex cause diarrhea in livestock and abortion of pregnant animals.	[14,115]
18	*Caltha palustris* Linn.	Ranunculaceae	Protoaneminin	Poison severity of this plant is low but this plant can be toxic, and ingesting large amounts of the plant’s leaves can lead to burning of the throat, vomiting, bloody diarrhea and gastric illness. Poisonous to human beings in mature stages.	[116,117]
19	*Cannabis sativa* Linn.	Cannabaceae	Cannabidiol, 9-tetrahydrocannabinol (THC)	Skin allergy.	[27]
20	*Capsicum chinense* Jacq.	Solanaceae	Capsaicin	Consuming excessive amounts may cause stomach irritation.	[17]
21	*Cassia occidentalis* Linn.	Fabaceae	Achrosin, aloe-emodin, emodin	Accidental intake of pods causes nausea, vomiting, restlessness, high fever, purging and ataxia in adult humans, whereas the accidental intake of seeds in childhood causes severe brain disease. In animals it causes gastroenteritis.	[14,118,119]
22	*Celtis australis* Linn.	Ulmaceae	Not reported	Regular consumption of leaves causes weakness and increase in body temperature in animals.	[14]
23	*Chelidonium majus* Linn.	Papaveraceae	Chelidonine	Ingestion causes the severe irritation of oral mucosa.	[14]
24	*Colchicum luteum* Baker	Liliaceae	Colchicine	Prolonged consumption may cause salivation with frothing in the mouth, colic, polydipsia, fetid diarrhea, dizziness and eventually death in a few cases.	[14]
25	*Commelina benghalensis* Linn.	Commelinaceae	n-octacosanol, n triacontanol, n-dotriacontanol	The plant is bitter in taste and after ingestion it causes stomach irritation in animals.	[14]
26	*Convallaria majalis* Linn.	Asparagaceae	Cardenolides	Neurotoxic, ingestion may cause cardiac dysrhythmia and hyperkalemia.	[27,60,120]
27	*Cuscuta reflexa* Roxb.	Cuscutaceae	Cuscutin, cuscutatin, beta-sitosterol, luteolin, bergenin and kaempferol	It causes vomiting, stomach ache, anorexia and purgation in animals, and its consumption can cause abortion in pregnant animals.	[14]
28	*Daphne oleoides* Schreb	Thymelaeaceae	Not reported	Berries and leaves consumption creates mouth sensation, nausea, vomiting, diarrhoea, restlessness, numbness and unconsciousness.	[14]
29	*Datura innoxia* Mill.	Solanaceae	Atropine	In humans the strong pungent smell of the leaves causes nausea and severe headaches. Contact with the leaves causes several skin problems. Unintentional consumption of the seeds by humans and animals causes dryness and sensation of the mouth and throat, stomach ache, numbness, anorexia, mydriasis, polydipsia and restlessness.	[14]
30	*Datura stramonium* Linn.	Solanaceae	Atropine	Accidental ingestion of the leaves or seeds by either humans or animals may cause drowsiness, dryness and sensation of the mouth and throat, bulging of the eyeballs, mydriasis, blurred vision, startling movements, convulsions, unconsciousness and finally death.	[14]
31	*Delphinium brunonianum* Royle	Ranunculaceae	Diterpenoid, alkaloids, Methyllycaco-ninite	Skin allergy.	[27]
32	*Digitalis purpurea* Linn.	Plantaginaceae	Digitoxin and Digoxin	Ingestion of the plant may cause nausea, vomiting, abdominal pain, excessive urination, abnormal heartbeats and finally death.	[17]
33	*Ephedra sinica* Stapf.	Ephedraceae	Ephedrine	Plant ingestion may cause nausea, vomiting, abdominal pain, hyperkalemia and cardiac dysrhythmias.	[17]
34	*Eupatorium adenophorum* Spreng.	Asteraceae	2-deoxo-2-(acetyloxy)-9-oxoageraphorone, 9-oxo-10, 11-dehydroageraphorone, 10Hβ-9-oxoageraphorone, and 10Hα-9-oxo-ageraphorone	Ingestion of the plant may cause coughing, increased respiratory effort and weight loss in horses.	[69,70]
35	*Gloriosa superba* Linn.	Colchicaceae	Colchicine	Every part of this plant is poisonous, especially the tuberous rhizome, and after ingestion it may cause nausea, abdominal pain, vomiting, numbness, burning in the throat and bloody diarrhea, which leads to dehydration.	[121]
36	*Heracleum canescens* Lindl.	Apiaceae	Furanocoumarins	Skin contact with furanocoumarins caused sensitization when exposed to sunlight or UV light. Furanocoumarins enter to the nucleus and binds with DNA and causes cell death and inflammation.	[72]
37	*Hedera nepalensis* K.Koch	Araliaceae	Saponins	When the skin comes in contact with the leaves it causes skin problems such as rashes and severe swelling in susceptible individuals. Consumption of leaves is poisonous for animals, causing paralysis and finally death.	[14]
38	*Hyoscyamus niger* Linn.	Solanaceae	Tropane alkaloids	Ingestion causes dry mouth, dysphonia, tachycardia, dysphagia, mydriasis, headache, urinary retention and confusion.	[27]
39	*Hypericum perforatum* Linn.	Hypericaceae	Hypericin, pseudohypericin, and hyperforin	Plant intake during flowering phases causes itching, photosensitization and inflammation of affected areas of skin, dry cough, trembling of limbs, extreme body pains, cold sweat and intense fatigue are some other symptoms after ingestion.	[14]
40	*Ichnocarpus frutescens* (L.) W.T. Aiton	Apocynaceae	Not reported	Consumption of leaves by animals’ causes indigestion, sour belching, vomiting and stomach irritation.	[14]
41	*Lantana camara* Linn.	Verbenaceae	Lantadenes	Jaundice, diarrhea, weakness, lethargy, loss of appetite, photosensitivity and hepatotoxicity in grazing animals.	[77]
42	*Melia azedarach* Linn.	Meliaceae	Tetranortriterpenes (meliatoxins)	Neurotoxin, gastrointestinal.	[27,79]
43	*Nerium indicum* Mill.	Apocynaceae	Oleandrin	Consumption of this plant causes mild to severe symptoms such as increased blood pressure and heart rate, sweating and vomiting. Its excessive consumption leads to heart attack and sudden death.	[14]
44	*Physalis minima* Linn.	Solanaceae	Solanine	Consumption of unripe berries causes abortion in pregnant animals.	[14]
45	*Prunus persica* (L.) Batsch	Rosaceae	Cyanide	Excessive consumption of the newly developed leaves affects brains and causes severe symptoms, i.e., seizures, loss of consciousness, abdominal pain, convulsions, choking, and finally death within a few hours in animals.	[14]
46	*Ranunculus arvensis* Linn.	Ranunculaceae	Protoanemonin	This plant may cause skin inflammation and injury of mucous membranes. The fresh leaf juice causes cracks, itching and sores in the skin of humans and animals.	[14,122]
47	*Ranunculus sceleratus* Linn.	Ranunculaceae	Protoanemonin	When the skin or mucosa comes in contact with the injured part of the plant, it causes itching and skin rashes and blisters. Poison ingestion may cause dizziness, nausea, vomiting, acute hepatitis, jaundice and finally paralysis.	[123,124]
48	*Rhamnus triquetra* Wall.	Rhamnaceae	Rhamnetin, quercitin and rhamnazin	Fruits and leaves of this species are highly toxic for livestock and excessive consumption affects the working ability of their brain resulting in loss of mental balance.	[14]
49	*Rhododendron campanulatum* D.Don	Ericaceae	Andromedotoxin	After ingestion of flowering buds and leaves by livestock it causes salivation, diarrhea, loss of energy and finally death.	[84,85]
50	*Ricinus communis* Linn.	Euphorbiaceae	Ricin	In humans, it causes mild to severe symptoms after ingestion, i.e., pain in throat, inflammation in eyes, high fever, profuse cold sweat, difficulty in swallowing, vomiting, diarrhoea, nausea, weakness, trembling of hands, inability to stand and finally death.	[17]
51	*Solanum xanthocarpum* Schrad. & H. Wendl.	Solanaceae	Solasonine and solamargine	After ingestion it causes headaches, nausea, vomiting, diarrhea, stomach ache, burning of the throat, itching, eczema, thyroid problems and pain and inflammation in the joints.	[96]
52	*Taxus baccata* Linn.	Taxaceae	Taxanes or Taxines, Taxol	Seeds and leaves are highly toxic, causing nausea, vomiting, abdominal pain bradycardia and respiratory muscle paralysis.	[125]

## 4. Role of Poisonous Plants to Cure Several Diseases

Plants have been used as a potential source of drugs since ancient times due to the presence of a large variety of bioactive compounds [126]. The presence of bioactive compounds in plants makes them important for the manufacture of therapeutic drugs. In recent years, more attention has been paid to the use of herbal medicine all over the world, which mainly includes traditionally used plants, due to which there has been a rapid increase in the research and revival of this traditional knowledge. For a long time, several compounds have been discovered in plants that can be used for human welfare, poisonous plants being one of them. In addition to plant toxicity, these toxic compounds have a long and colorful history of use for humans that have been documented in much of the world’s literature. These poisonous plants are practiced in folk medicine to treat diseases of humans and animals, with low doses being often beneficial, while overdose can induce toxicity [127] (Table 2). The right dosage of the plant’s bioactive compounds acts as medicine and differentiates it from the poison. Some poisonous plants are also rich in nutraceutical constituents and used in several traditional and modern pharmacopeia; hence, they are potential candidates for nutritional and medicinal prerequisites in emergency situations. The toxic compounds present in some plants, which is responsible for their toxicity for humans and other forms of life, are also used for other purposes, such as to treat several diseases, as pesticides and for food flavoring purposes. These toxic compounds are often part of the phenolics, alkaloids, tannins, saponins, glycosides and oxalates [15].

## 5. Conclusions and Future Prospective

There is scarce information available regarding the poisonous plants from the IHR, along with their toxicological profile, even though this review article has identified many poisonous plants from the region with recognized importance due to their toxic nature for humans and other animals, despite their renowned benefits for humans to cure several types of diseases. The toxicity of a plant depends on the presence of several chemical compounds and their concentration, as well as on other factors, i.e., temperature, rainfall, age of plants, plant dosage, growth stage, time of collection and nutrients in soil. Some plants are highly toxic, causing cardio- and neurotoxicity, increased heart rate, vomiting, abdominal cramps and diarrhea, and ultimately death. On the other hand, some poisonous plants can cause mild symptoms, such as fever, skin allergies, headaches and weakness.

Some poisonous plants of the IHR have received high attention from researchers, as well as the pharmaceutical (and other) industries due to their medicinal value, while some plants have received less attention for the reason that the exact mechanism of their toxicity is unknown. In this review, we have described in detail some poisonous plants found in the IHR, their toxicity, toxic compounds and their post-ingestion symptoms, which will be helpful as a guide for researchers, the pharmaceutical industry and toxicological studies. We also believe that this review will be helpful to increase the public knowledge and awareness about plant toxicity, which will help to further prevent poisoning and avoid public health issues.

Some of the poisonous plants of the IHR are being illegally harvested due to their high medicinal values and because of their over-exploitation they are categorized as threatened species. On the other hand, due to the toxicity of these plants to animals, these plants are wiped out by the local inhabitants, due to which the existence of these plants may be threatened in the future. Therefore, it is urgent need to develop conservation strategies for these plants as well as aware the people about their toxicity especially in children.

Further studies are needed towards to a better understanding of the detailed mechanism of action of these poisonous plants, as well as their role in curing a variety of diseases. More information about the toxic compounds of these plants could help develop a variety of drugs. On the other hand, the high demands of some poisonous plants have caused their overexploitation in their natural habitat and they are facing a high risk of extinction. Therefore, there is a need for a policy for the conservation of these poisonous plants, which can be achieved by encouraging farmers to cultivate them as, and along with their conservation, it will be helpful in protecting them as well as increasing the income of the farmers. It is also worth noting that there is a lack of data availability about the number of people and animals affected by poisonous plants. The availability of such data can be useful for the government, researchers and other agencies to raise people’s awareness about plant toxicity. Hence, there is a need to develop such a plan which gives information about the loss of humans and animals due to plant poisoning every year.

## Figures and Tables

**Figure 4:**
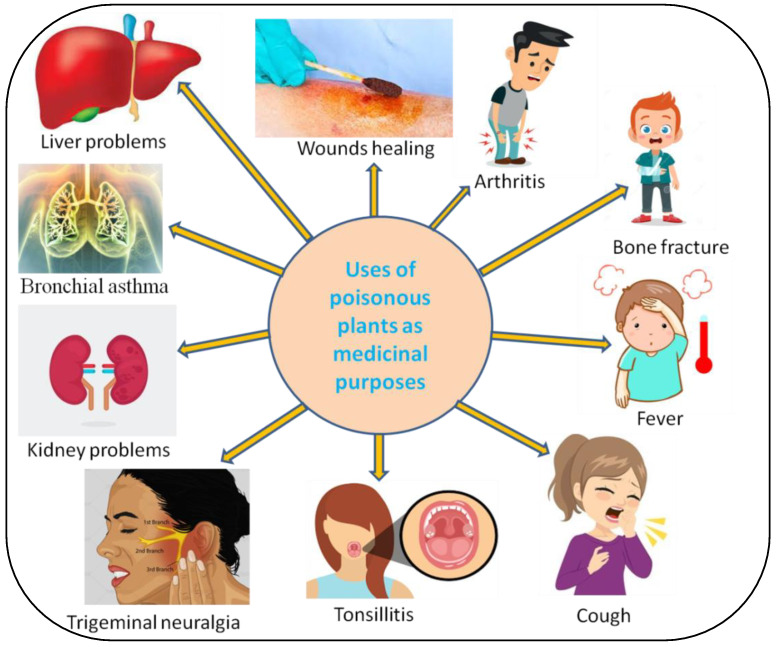
Uses of poisonous plants to cure several diseases.

**Table 2:** Medicinal uses of some poisonous plants (Figure 4).

S. No.	Poisonous Plants	Medicinal Properties	References
1	*Abrus precatorius* Linn.	Traditionally used to treat tetanus and to prevent rabies.	[32]
2	*Aconitum lethale* Griff.	Used to cure leprosy and arthritis, fever, rheumatism and boils.	[128,129,130,131,132,133]
3	*Aconitum chasmanthum* Stapf ex Holmes	Used in neuralgia, beurological rheumatism, as anthelminthic and as body tonic.	[134,135]
4	*Aconitum ferox* Wall. ex Ser.	Fever, digestive problems, leprosy, cholera inflammation and cuts, after detoxification of dried tubers by boiling in alcohol and used for fever, throat pain, tonsillitis, stomach ache and cheilitis.	[136,137,138,139]
5	*Aconitum hookeri* Stapf	Dried roots are used in diabetes and jaundice.	[140]
6	*Aconitum laeve* Royle	Used in kidney problems such as kidney stones, cold, cough, vomiting and diarrhea.	[141,142]
7	*Aquilegia pubiflora* Wall. ex Royle	Traditionally used in hepatitis, jaundice, wound healing, skin burns, circulatory and cardiovascular diseases.	[143,144,145]
8	*Arisaema triphyllum* (L.) Schott	Used in stomach ache and rheumatism, piles, dysentery. Tubers’ powder are used for the treatment of wound healing.	[146,147,148,149,150]
9	*Atropa belladonna* Linn.	Used to dilate the pupil of the eye, headache, peptic ulcer, menstrual symptoms, inflammation and motion sicknesss.	[151,152]
10	*Cannabis sativa* Linn.	Used for the treatment of various diseases, i.e., nausea, vomiting, diabetes, glaucoma, snake-bite, chronic pain, arthritis, bronchial asthma and cancer.	[153,154,155]
11	*Convallaria majalis* Linn.	Used in congestive heart failure and cardiomyopath	[156]
12	*Delphinium brunonianum* Royle	The dry and powdered rhizome is used in fever, headache stomach-ache, and cough.	[157]
13	*Hyoscyamus niger* Linn.	Used for the treatment for pupil dilating, urinary bladder relaxant, antisecretory, bronchodilating, anti-diarrheal properties, and as a spasmolytic, hypnotic hallucinogenic and sedative.	[158,159]
14	*Melia azedarach* Linn.	Stem and bark are used in gonorrhea, malaria and to expel parasitic worms, leaves are used for skin diseases such as scabies and for brushing teeth.	[160,161]
15	*Silybum marianum* (L.) Gaertn.	Used to treat liver and biliary disorders.	[162]
16	*Taxus baccata* Linn.	The taxol has high anticancer properties and used for cancer treatment.	[163]
